# Seroprevalence of SARS-CoV-2 Binding and Neutralizing Antibodies in Healthcare Workers during the Epidemic Peak in Referral Hospitals and Quarantine Sites: Saudi Arabia

**DOI:** 10.3390/v13071413

**Published:** 2021-07-20

**Authors:** Rowa Y. Alhabbab, Ahdab Alsaieedi, Abdullah Algaissi, Sara Almahboub, Rajaa M. Al-Raddadi, Omaima I. Shabouni, Rahaf Alhabbab, Abdulelah A. Alfaraj, Sawsan S. Alamri, Najwa D. Aljehani, Rwaa H. Abdulal, Mohamed A. Alfaleh, Turki S. Abujamel, Almohanad A. Alkayyal, Ahmad Bakur Mahmoud, Adel M. Abuzenadah, Anwar M. Hashem

**Affiliations:** 1 Vaccines and Immunotherapy Unit, King Fahd Medical Research Center, King Abdulaziz University, Jeddah 21589, Saudi Arabia; ralhabbab2@gmail.com (R.Y.A.); aalsaieedi@kau.edu.sa (A.A.); salmahboob@kau.edu.sa (S.A.); sawsan522@hotmail.com (S.S.A.); naljehani0053@stu.kau.edu.sa (N.D.A.); rowaahussain@gmail.com (R.H.A.); maalfaleh@kau.edu.sa (M.A.A.); tabujamel@kau.edu.sa (T.S.A.); 2 Department of Medical Laboratory Sciences, Faculty of Applied Medical Sciences, King Abdulaziz University, Jeddah 21589, Saudi Arabia; aabuzenadah@kau.edu.sa; 3 Department of Medical Laboratories Technology, College of Applied Medical Sciences, Jazan University, Jazan 45142, Saudi Arabia; aalgaissi@jazanu.edu.sa; 4 Medical Research Center, Jazan University, Jazan 45142, Saudi Arabia; 5 Department of Community Medicine, Faculty of Medicine, King Abdulaziz University, Jeddah 21589, Saudi Arabia; rmsalharbi@kau.edu.sa; 6 Ministry of Health, Jeddah 23325, Saudi Arabia; omimashabouni@gmail.com; 7 Department of Oral and Maxillofacial Surgery, King Fahad General Hospital, Jeddah 23325, Saudi Arabia; ralhabbab@moh.gov.sa; 8 Department of Phlebotomy, Blood Bank & Laboratory, King Fahad General Hospital, Jeddah 23325, Saudi Arabia; abbdulillah-al-fj@hotmail.com; 9 Department of Biochemistry, Faculty of Science, King Abdulaziz University, Jeddah 21589, Saudi Arabia; 10 Department of Biology, Faculty of Science, King Abdulaziz University, Jeddah 21589, Saudi Arabia; 11 Department of Pharmaceutics, Faculty of Pharmacy, King Abdulaziz University, Jeddah 21589, Saudi Arabia; 12 Department of Medical Laboratory Technology, University of Tabuk, Tabuk 71491, Saudi Arabia; aalkayyal@ut.edu.sa; 13 College of Applied Medical Sciences, Taibah University, Almadinah Almunwarah 42353, Saudi Arabia; abaMahmoud@taibahu.edu.sa; 14 Department of Medical Microbiology and Parasitology, Faculty of Medicine, King Abdulaziz University, Jeddah 21589, Saudi Arabia

**Keywords:** SARS-CoV-2, COVID-19 pandemic, anti-S Abs, seroprevalence, Jeddah, HCWs

## Abstract

Healthcare workers (HCWs) are at high risk for SARS-CoV-2 infection compared to the general population. Here, we aimed to evaluate and characterize the SARS-CoV-2 seropositivity rate in randomly collected samples among HCWs from the largest referral hospitals and quarantine sites during the peak of the COVID-19 epidemic in the city of Jeddah, the second largest city in Saudi Arabia, using a cross-sectional analytic study design. Out of 693 participants recruited from 29 June to 10 August 2020, 223 (32.2%, 95% CI: 28.8–35.8) were found to be confirmed seropositive for SARS-CoV-2 antibodies, and among those 197 (88.3%) had never been diagnosed with COVID-19. Seropositivity was not significantly associated with participants reporting COVID-19 compatible symptoms as most seropositive HCW participants 140 (62.8%) were asymptomatic. The large proportion of asymptomatic SARS-CoV-2 cases detected in our study demands periodic testing as a general hospital policy.

## 1. Introduction

In late December 2019, a cluster of atypical pneumonia was reported in people from Wuhan city in China. The etiological agent was later identified as a novel coronavirus (CoV) and subsequently named as severe acute respiratory syndrome coronavirus 2 (SARS-CoV-2) [1]. Given the explosive spread, the total of suffering countries, and the severity of the disease, SARS-CoV-2 was declared a global pandemic on 11 March 2020 by the World Health Organization (WHO) [2]. COVID-19 associated clinical symptoms range from asymptomatic to severe infection that might eventually lead to fatal outcomes [3,4,5,6]. Unfortunately, the actual number of SARS-CoV-2 infected individuals is currently unclear, at least in part due to the asymptomatic undocumented cases.

Healthcare workers (HCWs) are the most commonly exposed to SARS-CoV-2 due to their direct contact with confirmed as well as suspected COVID-19 cases. Moreover, if they get infected they could pose a risk to vulnerable patients, colleagues and families [7]. Therefore, the Saudi Ministry of Health (MOH) has implicated that HCWs with positive RT-PCR results are required to be isolated together with their close contacts, which mostly includes their fellow HCWs. High rates of infection among HCWs could disable the response of the healthcare system to the demand, particularly if the transmission rates increased among the frontline HCWs. To minimize this outcome, multiple strategies including offering continuous and easy access to SARS-CoV-2 screening tests, providing all the protective tools, and many other additional implicated measures in different settings were offered to provide proper patient care as possible. Nonetheless, it is important to monitor the prevalence of SARS-CoV-2 infection in HCWs to evaluate the infection rate and to determine the high-risk departments. Acknowledging the prevalence of SARS-CoV-2 infection among HCWs can also serve as a useful tool to avoid unnecessary quarantine and to properly plan healthcare resources [8].

**Figure 1:**
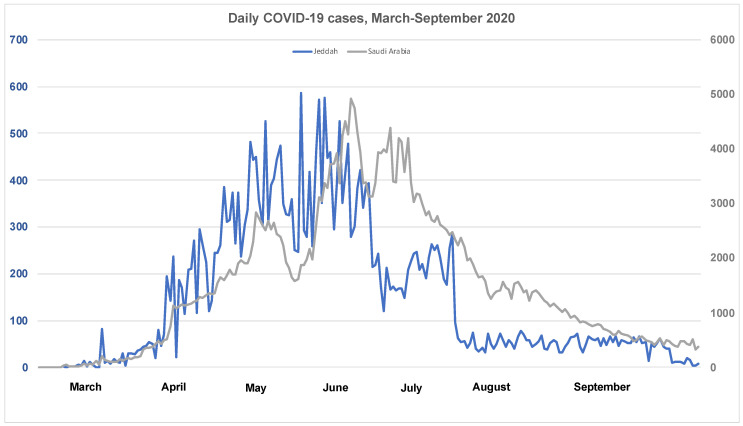
Daily cases of COVID-19 in Jeddah (March–September 2020). Data collected from the Ministry of Health Dashboard for COVID-19, Saudi Arabia (https://covid19.moh.gov.sa/, accessed on 30 May 2021).

This study aims to evaluate the seroprevalence of SARS-CoV-2 specific antibodies and to characterize the profile of these antibodies in HCWs from the largest reference hospitals and quarantine sites during the peak time of the pandemic in the city of Jeddah, which is the second largest city in Saudi Arabia, from 29 June to 10 August 2020 (Figure 1). We also report here the association of seropositivity with HCWs’ age, sex, previously reported SARS-CoV-2 infection, symptoms, and working sites during the same period.

## 2. Materials and Methods

### 2.1. Study Design, Population and Setting

This cross-sectional study was conducted from 29 June to 10 August 2020 in randomly selected HCWs from the 3 main referral hospitals in Jeddah as well as those working at 5 quarantine sites established for COVID-19 patients. The population presented here was divided into medical and administration staff. Participants were adults older than 17 years, working at the included hospitals and quarantine sites during the specified period. Those who reported absences from work for 30 days due to vacation or sabbatical during the study time frame, or who volunteered in any COVID-19-related clinical trials were excluded.

### 2.2. Ethical Approval

Signed informed consent forms were obtained from all participants, and institutional ethical approval was obtained from the Institutional Review Board (IRB) at the Ministry of Health, Saudi Arabia (Project number 1262). All research was performed in accordance with relevant guidelines and regulations.

### 2.3. Procedures

Recruiting flyers were distributed via hospital announcement systems or via emails. Volunteer participants were included based on inclusion and exclusion criteria that are described in Section 2.1. Each participant filled out a survey that included the following information: participant demographics (name, national ID, age, sex, marital state, etc.), professional information such as occupation and department, clinical data including any previous reported COVID-19 RT-PCR testing, previous confirmed infection with MERS-CoV and/or COVID-19, any history of COVID-19-related symptoms during the past six months (fever, headache, vomiting/nausea, diarrhea, anosmia, ageusia, and dry throat), and date of medical center visit if available. The data of participants with history of COVID-19 or MERS-CoV RT-PCR tests were obtained from the MOH system, and blood samples were collected from each participant for immunological testing. Samples were transported as soon as 3 h on the same day of collection, and serum were isolated and stored at −80 °C until use.

### 2.4. SARS-CoV-2 Antibodies Determination by ELISA

To determine the seroprevalence, we utilized an in-house ELISA test that we recently developed and validated [9] to measure SARS-CoV-2 IgM and IgG antibodies against the most immunogenic SARS-CoV-2 antigens, nucleocapsid protein (N) and spike glycoprotein (S). Briefly, commercial recombinant SARS-CoV-2 S1 subunit (amino acids 1–685) (Sino Biological, Beijing, China), and an in-house produced recombinant SARS-CoV-2 N protein were used to coat 96-well ELISA plates at 1 μg/mL and 4 μg/mL in phosphate-buffered saline (PBS), respectively, for overnight at 4 °C. Plates were then washed with PBS with 0.05% tween-20 (PBS-T). After blocking the plates with 5% skim milk in PBS-T buffer at 37 °C for 1 h, 1:100 diluted serum samples were incubated for 1 h at 37 °C and washed. Anti-IgG and anti-IgM HRP-conjugated antibodies (Jackson ImmunoResearch, West Grove, PA, USA) were then added for 1 h at 37 °C. Following incubation, plates were washed and 3,3′,5,5′-tetramethylbenzidine (TMB) substrate (KPL, Gaithersburg, MD, USA) was added for 30 min in the dark at room temperature, and the reaction was terminated by 0.16 M sulfuric acid. Absorbance was measured at 450 nm using the ELx808 microplate reader (BioTek, Winooski, VT, USA). Cutoff values for the ELISA were 0.4 for IgG N-ELISA, 0.55 for IgM N-ELISA, 0.17 for IgG S1-ELISA, and 0.3 for IgM S1-ELISA as previously determined [9].

### 2.5. Cells

African Green monkey kidney-derived Vero E6 cell line (ATCC, 1586) and Baby Hamster kidney BHK-21/WI-2 cell line (Kerafast, EH1011) were cultured and maintained in complete Dulbecco’s modified essential medium (DMEM) supplemented with penicillin (100 U/mL), streptomycin (100 μg/mL) and with 5 or 10% fetal bovine serum (FBS) in a 5% CO_2_ environment at 37 °C.

### 2.6. rVSV-ΔG/SARS-2-S*-Luciferase Pseudovirus Neutralization Assay

A pseudovirus based on the recombinant Vesicular stomatitis virus (VSV) bearing SARS-CoV-2 S protein (rVSV-ΔG/SARS-2-S*-luciferase pseudovirus) was generated and used in neutralization assay as we previously reported [10]. In brief, BHK21/WI-2 cells transfected with pcDNA expressing codon-optimized full-length SARS-CoV-2 S protein (GenBank accession number: MN908947) were infected with rVSV-ΔG/G*-luciferase (Kerafast, EH1020-PM) 24 h post-transfection. Supernatant containing rVSV-ΔG/SARS-2-S*-luciferase pseudovirus was then harvested 24 h post-infection and used for the neutralization assay. Here, heat-inactivated human serum samples at dilutions of 1:20 and 1:40 in DMEM containing 5% FBS were mixed with fixed amount of rVSV-ΔG/SARS-2-S*-luciferase pseudovirus that yields 2 × 10^4^ relative luciferase unit (RLU) and incubated at 37 °C, 5% CO_2_ for 1 h in duplicates. Then, 100 μL of the pseudovirus–serum mixtures were transferred into confluent Vero E6 cell monolayers grown in 96-well white plate with clear bottom and incubated at 37 °C in 5% CO_2_ humidified incubator for 24 h. Cell only control (CC) and virus control (VC) were included in quadruplicates with each assay run. After 24 h, cells were lysed and luciferase activity was measured using the Luciferase Assay System (Promega) according to the manufacturer’s instructions. Serum samples with ≥50% inhibition of luciferase activity at both dilutions compared to VC (virus only, no serum) were considered positive for nAbs against SARS-CoV-2. Inhibitory effect at each dilution was determined as follows: 100 − [(mean RLU from each dilution − mean RLU from CC)/(mean RLU from VC − mean RLU from CC) × 100].

### 2.7. Sample Size and Statistical Analysis

A total of 720 eligible HCWs were recruited in this study. SARS-CoV-2 antibodies seroprevalence, seropositivity of participants previously diagnosed with COVID-19, and seropositivity of participants reporting COVID-19-related symptoms were calculated with 95% CI as proportions. For categorical variables, we tested the association by using Chi-square or Fisher’s exact test, while for quantitative continuous variables we used Student’s *t*-test. To calculate the associated factors with the seroprevalence of SARS-CoV-2 antibodies we used univariable and multivariable analysis. Stepwise selection was used to include variables in the multivariable model, in which variables with *p* < 0.05 were added. Antibody levels association with age was calculated using non-linear regression determined by the LOESS method, while antibody levels’ correlation with the different groups was evaluated using two-tailed Mann–Whitney test. Analysis and calculations were conducted using GraphPad Prism 9 software and SPSS.

## 3. Results

### 3.1. Characteristics of the Study Participants

**Figure 2:**
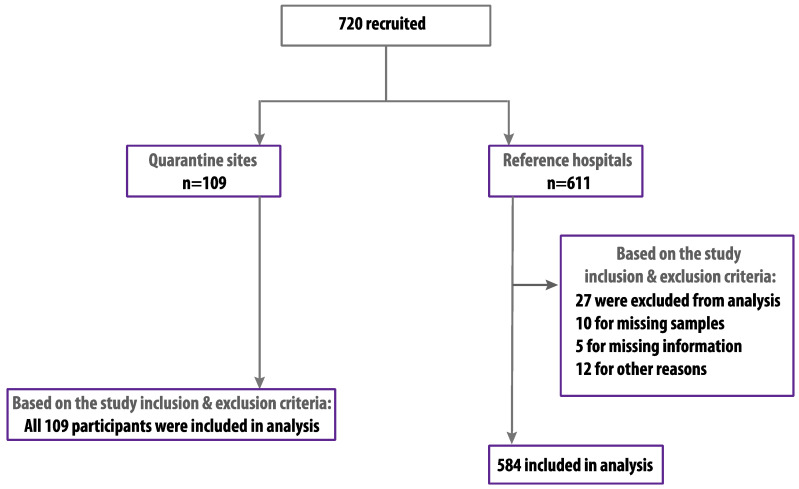
Study profile.

We randomly recruited 720 HCWs and hospital admin personnel between 29 June and 10 August 2020. Of these, 693 were eligible and included based on our inclusion and exclusion criteria with a participation rate of 96.3% (Figure 2). The study population included 346 (49.9%) males and 347 (50.1%) females with an average age of 36.5 ± 8.1 years in which 378 (54.6%) were older than 36 years. Among the participants, 557 (80.4%) were medical staff in direct contact with patients, in which 170 (30.5%) were covering COVID-19 care units, and 136 (19.6%) of the participants were non-medical staff, including administration and security personnel. Most of the participants 584 (84.3%) were from referral hospitals, and the rest 109 (15.7%) were from quarantine sites. The percentage of participants that were previously diagnosed with COVID-19 with confirmed RT-PCR results was 31 (4.5%), and only five of them were admitted to hospital, while only three participants (0.4%) were previously diagnosed with MERS-CoV. Among the study population, 276 (39.8%) reported one or more COVID-19-related symptoms during the months preceding this study (Table 1).

### 3.2. Seroprevalence of SARS-CoV-2 Antibodies in HCWs

**Figure 3:**
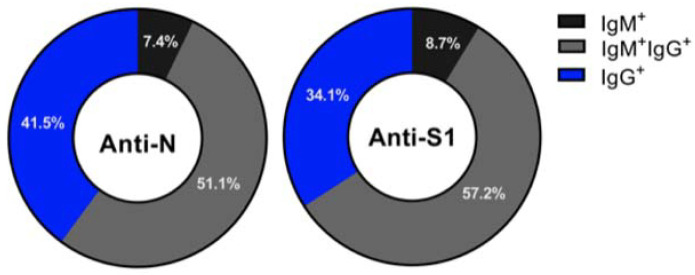
SARS-CoV2 antibody frequencies among seropositive participants. Parts of whole graphs represent the frequencies of seropositive subjects for antibodies against the SARS-CoV2 N protein (**left**) and S1 protein (**right**) according to their infection.

Seropositivity in this study was based on screening participants with N protein ELISA and confirming positive samples with S1-ELISA and pseudovirus neutralization assay. Only samples positive for anti-N and anti-S1 IgM and/or IgG with neutralizing antibodies (nAbs) were considered confirmed positives. A total number of 272 participants out of 693 (39.3%, 95% CI: 35.7–42.9) were seropositive for IgM and/or IgG antibodies against SARS-CoV-2 N antigen (Table 2). Among those, 20 (7.4%), 139 (51.1%), and 113 (41.5%) were seropositive for IgM only, IgM + IgG, and IgG only, respectively (Figure 3). Out of the participates who were seropositive for antibodies against N protein, 264 (97.1%) were also seropositive for anti-S1 antibodies, with the majority 151 (57.2%) being seropositive for both IgM and IgG, and only 23 (8.7%) were seropositive for IgM antibodies only, while the remaining 34.1% were seropositive for anti-S-IgG antibodies only (Figure 3). Only 223 (32.2%, 95% CI: 28.8–35.8) of those who were seropositive for anti-N and anti-S1 IgM and/or IgG showed nAb activity and were confirmed to be seropositive for SARS-CoV-2 (Table 2). Out of the confirmed positive cases, 125 (56.1%) were females, 127 (57.0%) were older than 36 years, 182 (81.6%) were HCWs, 207 (92.8%) were from referral hospitals, and 47 (21.1%) were working in COVID-19 units and in direct contact with COVID-19 patients (Table 2).

Out of the 31 participants who were previously diagnosed by RT-PCR with COVID-19, 26 (83.9%, 95% CI: 67.4–92.9) were found to be confirmed seropositive. All 26 individuals were seropositive for all antibodies including anti-N and -S1 IgM and/or IgG as well as nAbs, and all 5 seronegative persons were negative for all types of antibodies (Table 2). Interestingly, one of those 5 seronegative individuals was diagnosed twice with COVID-19 and was admitted to the hospital upon first infection, while the rest showed mild COVID-19-related symptoms only. Furthermore, all of those who were previously diagnosed with MERS showed nAbs for SARS-CoV-2. While 276 participants reported at least one COVID-19-related symptom, only 83 (30.1%, 95% CI: 25.0–35.7) were confirmed to be seropositive (Table 2).

### 3.3. Factors Association with Seropositivity

**Figure 4:**
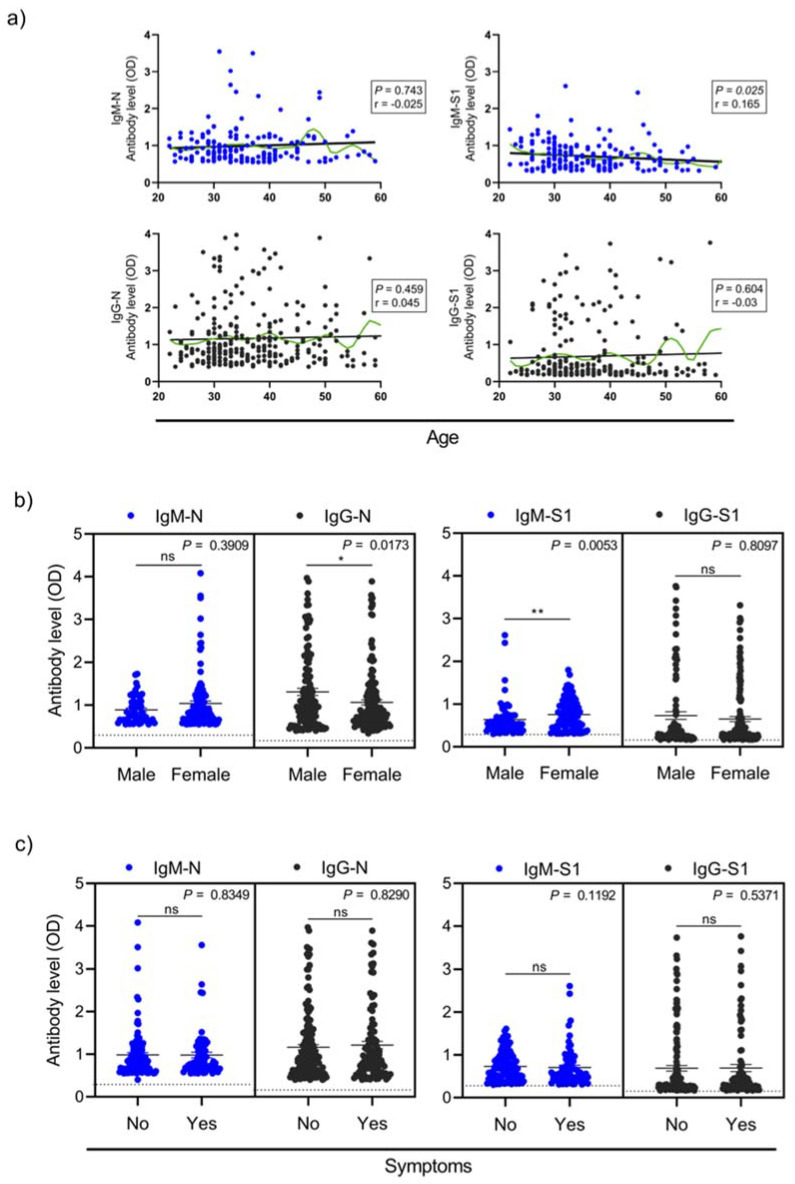
SARS-CoV2 antibody levels by age, gender and clinical variables. The levels of antibodies (optical density, OD) of IgM and IgG specific to SARS-CoV2 Spike protein (S1) and SARS-CoV2 Nucleocapsid protein (N) by age (**a**), gender (**b**), and symptoms (**c**). Data show only seropositive subjects for IgM-N, IgG-N, IgM-S1, and IgG-S1. Spearman test was applied to calculate the *p* values (*p*) and correlation coefficients (r) in (**a**), where the black line represents linear regression, and the green curve represents non-linear regression determined by the LOESS (locally estimated scatterplot smoothing) method. The center lines of the scatter graphs depict mean ± SEM; and two-tailed Mann–Whitney test was used to determine significant differences in the levels of antibodies between the groups in (**b**,**c**). * *p* < 0.05; ** *p* < 0.01.

Participants age and professional category had no significant relation with the existence of SARS-CoV-2 specific antibodies (Table 3). Nonetheless, we found that only S1-specific IgM antibody levels but not the other isotypes were higher in younger than older participants (*p* = 0.025) (Figure 4a). As shown in Table 3, participants’ gender did show significant association with confirmed seropositivity, with males having lower odds of being seropositive (adjusted OR: 1.59, 95% CI: 1.14–2.23; *p* = 0.006). The levels of anti-N IgG were higher in male than female participants (*p* = 0.0173), while anti-S1 IgM were greater in female compared to male subjects (*p* = 0.0053) (Figure 4b). Although the univariable logistic regression analysis showed some evidence that those not working at COVID-19 units had higher odds of showing seropositivity (OR: 0.75, 95% CI: 0.51–1.09), 47 (27.7%) out of the 170 participants working in COVID-19 units had confirmed seropositive results (Table 3). As expected, strong association was observed between reporting previous COVID-19 infection and seropositivity (Table 3).

The majority of the confirmed seropositive HCWs (62.8%, 140/223) were asymptomatic, and only 83 out of 223 (37.2%) experienced symptoms (Table 3). No significant association was found between experiencing COVID-19-related symptoms in the past months with seropositivity for the different antibody isotypes against N or S1 antigens (Figure 4c). Confirmed seropositivity was not significantly associated with reporting any COVID-19-related symptoms within the past months (OR: 0.84, 95% CI: 0.60–1.17, *p* = 0.334) (Table 3). The main COVID-19-related symptoms reported by confirmed seropositive individuals were headache, dry throat, diarrhea, fever, ageusia, anosmia, and nausea/vomiting (Table 4).

## 4. Discussion

This study is reporting the seroprevalence of SARS-CoV-2 antibodies among a representative group of HCWs and hospital admin personnel in the city of Jeddah, Makkah province, Saudi Arabia. Here, we have documented that 32.2% (95% CI: 28.8–35.8) of HCWs and hospital admin personnel from the largest referral hospitals and quarantine sites developed detectable levels of antibodies against SARS-CoV-2. Study participants in this study were recruited between 29 June and 10 August 2020, which was during the peak of the COVID-19 epidemic in Saudi Arabia (Figure 1).

Although national SARS-CoV-2 seroprevalence in HCWs between 20 and 30 May 2020 in Saudi Arabia has been previously reported [11], the percentage was very low (2.37%) compared to our results. Nonetheless, seroprevalence in HCWs from Makkah province was reported to be the highest (6.31%) compared to other regions in Saudi Arabia [11]. Another study also reported 6.37% seroprevalence in HCWs in the city of Makkah in samples collected between June and July 2020 [12]. This difference between these studies and our report is mostly due to the difference in the data collection timeframe in which the previous studies were conducted when the whole country was under complete or partial lockdown, compared to our study, which was performed during the peak of the COVID-19 outbreak. Given the low numbers of positive RT-PCR cases reported among the HCWs in this study, and the previously reported low seroprevalence in HCWs in previous reports [11,12], the seroprevalence found here was higher than what we expected. Nevertheless, it is consistent with the increased level of confirmed and reported COVID-19 cases by MOH during the same period, and it could be due to the likelihood of a large number of patients admitted at the studied referral hospitals and quarantine sites.

The high rates of confirmed seropositive HCWs who had never been diagnosed with COVID-19 in the past (88.3%, 197/223) or those who were asymptomatic (62.8%, 140/223) clearly suggest that large numbers of infected individuals could be spreading the infection silently [13]. Nonetheless, no significant difference was observed between seropositivity in HCWs who are in direct contact with patients and admin personnel in hospitals and quarantine sites samples in this study, suggesting that some exposure could have happened outside healthcare facilities and quarantine sites. While most participants previously diagnosed with COVID-19 (83.9%) had detectable levels of nAbs, 16.1% of them were lacking any sort of antibodies against SARS-CoV-2, suggesting that not all recovered individuals might be protected from re-infection. The relatively high seroprevalence rate seen in our data indicates that HCWs are at high risk while serving their community. It also highlights the importance of following precautionary measures and reinforcement of a periodic screening program for HCWs to reduce the disease transmission within hospitals and quarantine sites to the minimal level [14].

Seropositivity was not associated with HCWs who experienced COVID-19-related symptoms. However, anosmia and ageusia were previously noted as the most associated symptoms in seropositive HCWs in Spain [2]. By contrast, in our study, we found that headache, dry throat and diarrhea were the most commonly reported symptoms among seropositive HCWs.

It is worth mentioning that we combined different assays including ELISA to detect IgM and IgG antibodies against both SARS-CoV-2 N and S1 proteins as well as a neutralization assay in our study to have a robust seroprevalence estimation and accurate identification of seropositive individuals [15,16]. Our testing algorithm also helps in detecting acutely infected individuals as well as convalescents, hence increasing the detection sensitivity. Using such an algorithm, we found that 39.3%, 38.1% and 32.2% of the tested participants had anti-N IgM ± IgG, anti-S1 IgM ± IgG and nAbs, respectively. While this might indicate that not all individuals with nAbs in this study produced IgM and/or IgG against N and S1 proteins, one cannot ignore the fact that some might have no or low antibody responses [17,18,19,20,21,22]. 

Here, we report that the seroprevalence of SARS-CoV-2 in HCWs and admin personnel working in major referral hospitals and COVID-19 quarantine sites in the second largest city in Saudi Arabia was higher than previously reported. Most importantly, we found that most of the seropositive HCWs have never been diagnosed with COVID-19 or tested positive for COVID-19, or experienced any COVID-19-related symptoms in the past. Furthermore, a good proportion of those who were known to be infected with SARS-CoV-2 in the past lacked any sort of antibodies against SARS-CoV-2. While these results show that a higher rate of some immunity might exist and could help in achieving herd immunity faster and/or reduce the need for multiple vaccine doses, our report clearly highlights the importance of implementing periodic screening programs by combining serological assays and molecular testing for all HCWs in addition to strict adherence to infection-control measures to reduce the burden of SARS-CoV-2 spread in hospitals.

## Figures and Tables

**Table 1:** Characteristics of study participants.

Characteristic	Category	Number of Participants *n* (%)
Overall		693
Gender	Male	346 (49.9%)
	Female	347 (50.1%)
Age ^a^		36.5 ± 8.1
Professional category	HCW	557 (80.4%)
	Non HCW	136 (19.6%)
Site	Referral hospitals	584 (84.3%)
	Quarantine sites	109 (15.7%)
Working in COVID-19 unit	Yes	170 (24.5%)
	No	523 (75.5%)
Diagnosed previously with COVID-19	Yes	31 (4.5%)
	No	662 (95.5%)
Diagnosed previously with MERS	Yes	3 (0.4%)
	No	690 (99.6)
Reported any COVID-19 related symptoms	Yes	276 (39.8%)
	No	417 (60.2%)

^a^ Mean ± SD.

**Table 2:** Overall proportion of seropositive HCWs.

		Total (*n*)	Seropositivity *n* (%; 95% CI)		
			Anti-N IgM ± IgG	Anti-S1 IgM ± IgG	nAbs
Overall		693	272 (39.3%; 35.7–42.9)	264 (38.1%; 34.5–41.7)	223 (32.2%; 28.8–35.8)
Gender	Male	346	118 (34.1%; 29.3–39.3)	114 (33.0%; 28.2–38.1)	98 (28.3%; 23.8–33.3)
	Female	347	154 (44.4%; 39.2–49.6)	150 (43.2%; 38.1–48.5)	125 (36.0%; 31.2–41.2)
Age group	>36	378	155 (41.0%; 36.2–46.0)	150 (39.7%; 34.9–44.7)	127 (33.6%; 29.0–38.5)
	<36	315	117 (37.1%; 32.0–42.6)	114 (36.2%; 31.1–41.6)	96 (30.5%; 25.7–35.8)
Professional category	HCW	557	225 (40.4%; 36.4–44.5)	221 (39.7%; 35.7–43.8)	182 (32.7%; 28.9–36.7)
	Admin	136	47 (34.6%; 27.1–42.9)	43 (31.6%; 24.4–39.9)	41 (30.2%; 23.1–38.3)
Site	Referral hospitals	584	246 (42.1%; 38.2–46.2)	238 (40.8%; 36.8–44.8)	207 (35.5%; 31.7–39.4)
	Quarantine sites	109	26 (23.9%; 16.8–32.6)	26 (23.9%; 16.8–32.6)	16 (14.7%; 9.2–22.5)
Working in COVID-19 unit		170	55 (32.4%; 25.8–39.7)	55 (32.4%; 25.8–39.7)	47 (27.7%; 21.5–34.8)
Diagnosed previously with COVID-19		31	26 (83.9%; 67.4–92.9)	26 (83.9%; 67.4–92.9)	26 (83.9%; 67.4–92.9)
Diagnosed previously with MERS		3	3 (100%; 43.9–100)	3 (100%; 43.9–100)	3 (100%; 43.9–100)
Reported any COVID-19 related symptoms		276	107 (38.8%; 33.2–44.6)	101 (36.6%; 31.1–42.4)	83 (30.1%; 25.0–35.7)

**Table 3:** Univariable and multivariable analysis of factors associated with seropositivity.

Characteristic	Category	Seronegative	Seropositive	*p* Value	Univariable Analysis	Multivariable Analysis	
		(*n* = 470)	(*n* = 223)		OR (95% CI)	OR (95% CI)	*p* Value
Gender ^a^	Male Female	248 (52.8%) 222 (47.2%)	98 (43.9%) 125 (56.1%)	0.030 ^b^	1 1.41 (1.03–1.95)	1 1.59 (1.14–2.23)	0.006 ^e^
Age ^c^		36 ± 8.1	37 ± 8.0	0.860 ^d^	1.04 (0.75–1.44)		
Professional category ^a^	HCW Admin	375 (79.8%) 95 (20.2%)	182 (81.6%) 41 (18.4%)	0.572 ^b^	1 1.12 (0.75–1.70)	1 1.03 (0.67–1.59)	0.877 ^e^
Working in COVID-19 unit ^a^	Yes No	123 (26.2%) 347 (73.8%)	47 (21.1%) 176 (78.9%)	0.145 ^b^	0.75 (0.51–1.09) 1		
Diagnosed previously with COVID-19 ^a^	Yes No	5 (1.1%) 465 (98.9%)	26 (11.7%) 197 (88.3%)	<0.0001 ^b^	12.27 (5.04–36.7) 1	13.4 (5.48–40.4) 1	<0.0001 ^e^
Diagnosed previously with MERS ^a^	Yes No	0 (0%) 470 (100%)	3 (1.3%) 220 (98.7%)	0.012 ^b^			
Reported any COVID-19 related symptoms ^a^	Yes No	193 (41.1%) 277 (58.9%)	83 (37.2%) 140 (62.8%)	0.334 ^b^	0.84 (0.60–1.17) 1		

^a^ *n* (column percentage); ^b^ Chi-squared test; ^c^ Mean ± SD; ^d^ *t*-test; ^e^ Wald test.

**Table 4:** Symptoms association with confirmed seropositivity.

		Total (*n* = 693)	Seropositive ^a^ (*n* = 223)	Seronegative ^a^ (*n* = 470)	*p* Value ^b^
Headache	Yes No	182 (26.3%) 511 (73.7%)	59 (26.5%) 164 (73.5%)	123 (26.2%) 347 (73.8%)	0.936
Dry throat	Yes No	149 (21.5%) 544 (78.5%)	40 (17.9%) 183 (82.1%)	109 (23.2%) 361 (76.8%)	0.116
Diarrhea	Yes No	91 (13.1%) 602 (86.9%)	25 (11.2%) 198 (88.8%)	66 (14.0%) 404 (86.0%)	0.303
Fever	Yes No	61 (8.8%) 632 (91.2%)	21 (9.4%) 202 (90.6%)	40 (8.5%) 430 (91.5%)	0.694
Ageusia	Yes No	47 (6.8%) 646 (93.2%)	19 (8.5%) 204 (91.5%)	28 (6.0%) 442 (94%)	0.210
Anosmia	Yes No	44 (6.4%) 649 (93.6%)	18 (8.1%) 205 (91.9%)	26 (5.5%) 444 (94.5%)	0.200
Nausea/vomiting	Yes No	43 (6.2%) 650 (93.8%)	11 (4.9%) 212 (95.1%)	32 (6.8%) 438 (93.2%)	0.339

^a^ *n* (column percentage); ^b^ Chi-squared test.

## Data Availability

Datasets produced and analyzed in this study are available from the corresponding author upon reasonable request.
